# Community-oriented primary care for National Health Insurance in South Africa

**DOI:** 10.4102/phcfm.v14i1.3243

**Published:** 2022-02-24

**Authors:** Shabir Moosa

**Affiliations:** 1 Department of Family Medicine and Primary Care, Faculty of Health Sciences, University of the Witwatersrand, Johannesburg, South Africa; 2 WONCA Africa, Johannesburg, South Africa

**Keywords:** family medicine, family physician, community oriented primary care, primary health care, universal health coverage, national health insurance, complexity theory, complex adaptive systems, community practice

## Abstract

This is a report on Chiawelo Community Practice (CCP) in Ward 11, Soweto, South Africa, a community-oriented primary care (COPC) model for National Health Insurance (NHI) in South Africa, developed by a family physician. A shift to capitation contracting for primary health care (PHC) under NHI will carry risk for providers – both public and private, especially higher number of patient visits. Health promotion and disease prevention, especially using a COPC model, will be important. Leading the implementation of COPC is an important role for family physicians in Africa, but global implementation of COPC is challenged. Cuba and Brazil have implemented COPC with panels of 600 and 3500, respectively. The family physician in this report has developed community practice as a model with four drivers using a complex adaptive system lens: population engagement with community health workers (CHWs), a clinic re-oriented to its community, stakeholder engagement and targeted health promotion. A team of three medical interns: 1 clinical associate, 3 nurses and 20 CHWs, supervised by the family physician, effectively manage a panel of approximately 30 000 people. This has resulted in low utilisation rates (less than one visit per person per year), high population access and satisfaction and high clinical quality. This has been despite the challenge of a reductionist PHC system, poor management support and poor public service culture. The results could be more impressive if panels are limited to 10 000, if there was a better team structure with a single doctor leading a team of 3–4 nurse/clinical associates and 10–12 CHWs and PHC provider units that are truly empowered to manage resources locally.

## Introduction

Doctors trained in family medicine are a scarce resource, but African countries cannot afford to not invest in it. They are not only highly trained as clinicians but also as team-players: capacity builders, clinical trainers, leaders of clinical governance and advocates of community-orientation. It is challenging to show evidence of the value of family physicians in Africa because of poor political will and opposition on the ground.^1^ This is a report of the significant and unique work carried out in developing Chiawelo Community Practice (CCP) in Soweto, South Africa, as a community-oriented primary care (COPC) model for Universal Health Coverage (UHC) in South Africa, called National Health Insurance (NHI).

Whilst family physicians strongly tout the value of COPC there has been little evidence of effectiveness, even in the African context. An older systematic review on COPC and its effectiveness, showed that most COPC implementation does not use the complete COPC model described by the Karks (linking personal care to public health), with evidence of effectiveness lacking.^2^ Even public health practitioners focus only on public health in COPC.^3^

Cuba shows high quality outcomes with family physician–nurse pairs using COPC for populations of 600, but the large number of family physicians required in Africa appears too ambitious.^4^ Brazil has shown value with young doctor–nurse pairs implementing COPC with larger panels of 3500.^5^

South Africa implemented primary health care (PHC) re-engineering as part of preparing for NHI.^6^ Inspired by Brazil they planned ward-based outreach teams (WBOTs) of two professional nurses and six community health workers (CHWs) taking care of 7500 people, covering all minor ailments. Implementation quickly morphed into a verticalised programme with one 2-year trained enrolled nurse looking after 10–15 CHWs (each with 150 families) in a nebulously defined community and struggling to link patients to professional nurses in clinics.^7^

National Health Insurance plans include strategic purchasing, separating purchasers (NHI Fund) from providers (e.g. clinics), and contracting private general practitioners (GPs). Funding will change from budgets for clinics (and fee-for-service for GPs) to capitation. I did a study on GPs across South Africa exploring their response to a hypothesised capitation contract for a 10 000-size panel assuming three visits per person per year (i.e. 120 patients a day for 250 working days in a year, half for preventive care and half for acute/chronic care). I asked GPs to respond to their staffing changes, costs and possible charge. General practitioners envisaged needing 8–14 staff, mostly nurses. Their pricing point turned out to be very close to public sector costs. When asked about the risks of capitation (and ways of mitigating those risks) three issues emerged: unreliable contractor (needing a strong contract), high patient utilisation (needing health promotion) and larger organisations (needing management skills).^8^ A key question was how we could implement prevention to reduce utilisation rates, whilst improving quality of care. Community-oriented primary care, as described by the Karks in South Africa in the 1940s, was the way to do it.

The Karks had two innovations. First was the Community Health Centre (CHC), with two doctors caring for ±30 000 people using task-sharing with nurses and medical aides. This was an important but curative exercise. Their second innovation was COPC. The literature is littered with nuances, many missing the link between personal care and public health and becoming reductionist, as with WBOTs. I approached it using a complexity theory lens, specifically identifying a few drivers, to make it simpler to implement. The boundaries were a doctor-led PHC team providing as comprehensive PHC as possible to a defined population, initially set at 10 000.

## Family physician contribution

**FIGURE 1:**
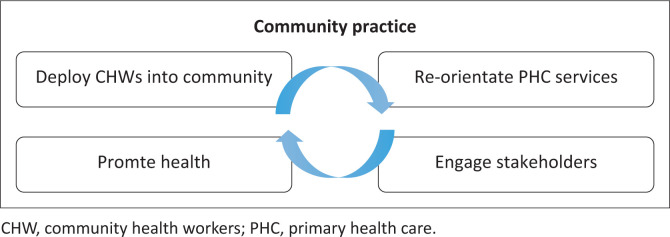
Community practice model.

In 2014, I implemented CCP using four drivers: population engagement with CHWs, re-oriented primary care at the clinic, stakeholder engagement and targeted health promotion (Figure 1). The CCP was expected to be a small complex adaptive system led by a family physician and a model that could be emulated by GP-led teams under NHI.

Chiawelo CHC (CCHC), in Ward 11 of Soweto, is one of five major CHCs in Soweto serving ±150 000 people in five wards directly. However, doctors and nurses work separately in strongly verticalised services. The CCP, located in one of CCHC’s 11 buildings, started with 20 CHWs and 1 enrolled nurse team leader paid via research funds. Chiawelo community practice is part of the CCHC but ‘led’ by a family physician, albeit limited by public service constraints.

### Deploying community health workers into the community

**FIGURE 2:**
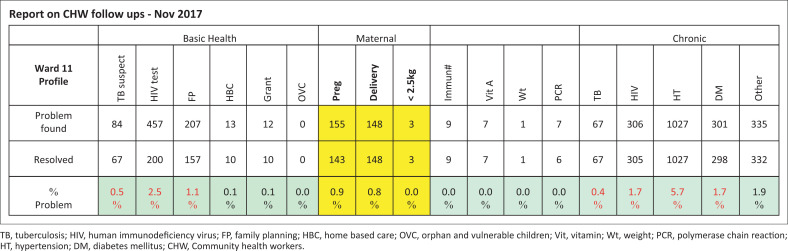
Report on problem list for Ward 11 (with percent of problems by population of 18 076 people registered into Chiawelo community practice).

Fifteen CHWs were deployed to streets in Ward 11, developing a street summary (a ‘census’) in late 2014 and then using WBOT Household Registration Forms to collect information from consenting families in 2015. Information included household details (including housing), family members and household illness/health risk issues (human immunodeficiency virus [HIV], tuberculosis [TB], pregnancy, etc.). Community health workers collated this into a community profile. The ‘census’ of Ward 11 as at December 2017 showed a population of 21 602. Community health workers had registered 18 076 people/4253 families (4.3 persons per family), mostly families of grandmothers, problem children and orphaned grandchildren, with extra rooms and yard shacks rented to migrants. Community health workers’ data showed 3139 (mostly health) problems as identified by the registration form. Community health workers used a ‘problem’ list to follow up on and resolve 89% of problems by 2017 (Figure 2). There was a programme of CHW training, eventually settling into once-a-week morning sessions. Unfortunately, CCP was forced by managers to expand to Wards 11, 12, 15, 16 and 19 in 2018 covering ±30 000 people, disrupting overall supervision, as many CHWs appointed via politically connected non-government organisations came with a poor work ethic. Ward 11 CHWs still function well.

### Re-orientating primary health care services to community

‘Registered’ patients are invited by CHWs to go directly to CCP as their ‘NHI clinic’, with a phone booking option. Services are limited to acute and chronic care as nursing managers refuse to provide CCP with nurses, consumables and support for immunisation, family planning and antenatal care. Chiawelo community practice was managed by five CHWs from 2014 to 2017 (one coordinator, two receptionists and two doing vitals). This was disrupted in 2018 when all CHWs had to rotate weekly in groups through CCP to assist with filing and vitals. This continues to date, compromising administration. In 2019 a professional nurse started coordinating CCP with an enrolled nurse doing vitals, with help from CHWs. When a patient arrives at CCP she or he is validated by Household Registration Forms kept in a folder for each CHW. A family folder is made up (with individual patient files and a CHW file [containing the filled household registration form]), with CHW name on all files. All patients are seen by three interns (rotating weekly in CCP for orientation to COPC as part of their family medicine block) and a clinical associate for continuity, all supervised by a family physician.

### Engage stakeholders

Community health workers mapped out stakeholders and social assets. Chiawelo community practice was launched on regular one-to-one and group discussions with a variety of stakeholders in the community (councillors, ward committees, parliamentary office, etc.) health sector (CCHC, private GPs, etc.) and government sectors (schools, police, etc.). Strong stakeholder engagement forced district managers to incorporate CCP by mid-2016 when research funding ran out. An annual stakeholder meeting reviews CCP and develops community priorities. Poverty, unemployment and drug abuse remain the big problems in the community. Bringing leaders together monthly has been challenging with their competing priorities. Patient group leaders have become the main participants of monthly community stakeholder meetings until the coronavirus disease 2019 (COVID-19) disruption in March 2020.

### Targeted health promotion

We apply health promotion and prevention at many levels. Community health workers address social determinants of health as primordial prevention (by linkages to various resources) and educate on risky behaviours as primary prevention, for example, smoking, sexual behaviour and so on. They also do secondary prevention or screening for disease as part of their process of visits. Community health workers in Ward 11 have set up five health clubs (with stretching and strengthening, some aerobics and then social support/health literacy discussions). Community health workers also visit schools to do screening and education. Community health workers also addressed drug abuse in campaigns and school dialogues. We encourage clinicians to write instructions to CHWs in CHW files passed to CHWs afterwards. Monthly stakeholder engagement is important for advocacy in health promotion. A chronic care flowchart supports chronic disease care and quality improvement in tertiary and quaternary prevention. Community health workers also carry out medicine delivery and education bimonthly with chronic patients.

All this has translated into CCP results, despite the challenges. Patients are not as happy now as they used to be because of the larger panel size, but CCP still functions differently from CCHC. Chiawelo Community Practice utilisation hovers at 0.67 visits per person per year, much less than public or private averages. An unpublished study of CCPs Ward 11 population in 2018 showed very high use of CCP strongly correlated with expressed need. Chiawelo Community Practice accessibility and satisfaction were very high compared with CCHC. Community health workers reduced patient visits substantially. An unpublished audit of chronic care showed much better CCP records and guidelines adherence than CCHC. An unpublished study showed CCP costs at a quarter of public service expenditure per capita in Johannesburg.

## Conclusion

Chiawelo Community Practice, as a model of care developed and supervised by a family physician, has shown value, even being used by the South African National Treasury to model NHI capitation contracting. With political will, systems theory and respect for professionalism, doctors trained in family medicine (and COPC) can provide UHC in South Africa at low cost when they lead an empowered decentralised PHC team caring for 10 000 people.
